# Implementation of Two-Dimensional Polycrystalline Grains in Object Oriented Micromagnetic Framework

**DOI:** 10.6028/jres.114.005

**Published:** 2009-02-01

**Authors:** J. W. Lau, R. D. McMichael, M. J. Donahue

**Affiliations:** Metallurgy Division, National Institute of Standards and Technology, Gaithersburg, MD 20899; Center for Nanoscale Science and Technology, National Institute of Standards and Technology, Gaithersburg, MD 20899; Mathematical and Computational Sciences Division, National Institute of Standards and Technology, Gaithersburg, MD 20899

**Keywords:** exchange coupling, magnetocrystalline anisotropy, micromagnetics, polycrystal

## Abstract

In response to the growing need for a more accurate micromagnetic model to understand switching phenomenon in nanoscale magnets, we developed the capability to simulate two-dimensional polycrystalline grains using the Object Oriented Micromagnetic Framework (OOMMF). This addition allows users full flexibility in determining the magnetocrystalline anisotropy and axe in each grain as well as the inter- and intragranular exchange coupling strength.

## 1. Introduction

Magnetic components in virtually all technological applications are polycrystalline due to cost constraints. Various magnetic components have been modeled with micromagnetics, largely as a single crystal with little or no magneto-crystalline anisotropy, The assumption is that when averaged over the entirety of the simulated dimensions, orientational dependence of anisotropy from randomly oriented grains will cancel out. This assumption has been valid for magnets of micron and sub-micron dimension for a long time. However, for smaller parts in the 100 nm range, it has been shown experimentally that the exact nature of grain orientation and distribution plays a crucial role in magnetization reversal behavior Refs. [1, 2]. In response to the growing need to model the effect of grains on switching dynamics, we have expanded OOMMF’s capability to model 2D through grains derived from Voronoi diagrams.

## 2. Usage

To use polycrystalline OOMMF, a Voronoi diagram must first be generated. The Voronoi diagram output is then read into an OOMMF micromagnetic input format (MIF) file. The grain-specific magneto-crystalline anisotropy axes data can be visualized and/or saved through the OOMMF mmDisp viewer. The details of each step are summarized below. Other OOMMF specific references, such as Oxs child classes, can be found in the OOMMF User’s Guide [3], available online at http://math.nist.gov/oommf/.

### A. Generating Grain Maps

The executable named voronoi is used to generate a random 2D grain map in the PPM (portable pixmap) bitmap image format. The command line switches −*x*, −*y*, and −*g* specify the number of pixels along the *x* and *y* directions and the number of grains, respectively. The executable defaults to a grain map of 512 pixels × 128 pixels and 2000 grains. When imported into a MIF file, the grain map will be automatically resized to fit the simulation dimensions; however it is recommended that the number of pixels along the *x* and *y* directions in the grain map closely match the number of simulation cells specified in the MIF file so that resizing errors may be minimized.

The grain maps produced by the executable voronoi are colored in a red-blue-green combination scheme, such that each grain has a unique 6 character designation in the form #rrggbb. Each of the 6 positions in rrggbb can take a hex digit value (i.e, 0–9 or A–F). The theoretical maximum number of grains that can be colored this way is therefore 16^6^ = 2^24^ (24-bit color). The optional command line switches -nored, -nogreen, -noblue may be used to restrict the output color space; this can be useful for rogue grain selection, as described below.

In the OOMMF simulation, the grains as described by the grain map are projected through the film. A full three dimensional simulation is possible using a single (two dimensional) grain map with the caveat that each grain traverses the entire thickness, orthogonal to the defined view-plane. On the other hand, it is possible to define multilayers with a unique grain map associated with each layer. In the case of a single layer material, the 2D through-grains assumption may be a reasonable approximation if the layer thickness is comparable to the grain size.

### B. MIF File Specifications

Appendices A and B present two examples of MIF files for polycrystalline samples. Appendix A is for a material with uniaxial magneto-crystalline anisotropy; a somewhat more complicated example involving cubic anisotropy is shown in Appendix B.

In both cases, the MIF files begin with the line

# MIF 2.2

which is the signature string for a MIF version 2.2 file. Some of the functionality supporting polycrystalline materials is new with version 2.2.

The grain map ( rect.ppm), where each grain is associated with a unique color, is parsed by the geometric volume interpreter, Oxs_ImageAtlas:


Specify Oxs_ImageAtlas:world [subst {
 xrange {0 $length}
 yrange {0 $width}
 zrange {0 $thick}
 viewplane xy
 image $grain_map
 colorfunction auto
 matcherror 0.0
}]


The xrange, yrange, zrange parameters specify the simulation dimensions in meters, while the viewplane xy parameter tells the atlas to orient the grain map (as specified by the image parameter) with the *xy*-plane.

In pre-2.2 versions of MIF, the Oxs_ImageAtlas object required an explicit list of colors and region names. However, starting with MIF 2.2, the colorfunction auto option may be used instead to automatically assign distinct logical regions to each color occuring in the image. The assigned region names have the form #rrggbb, where rrggbb represent the 24-bit color of each grain as described earlier. In general, the user does not need to know the specific region names; the fact that each color (and hence grain) is assigned to a distict region is sufficient to assign different easy axis orientations, magneto-crystalline anisotropy and exchange coupling to each grain. The one exception is the case where one wants to assign particular properties to a particular grain; this is the case with “rogue grain” assignment, which is discussed below.

In Appendix A, a material with uniaxial anisotropy is modeled that has uniform *K*1, but with each grain having an easy axis randomly selected within the confines of a texture cone, that is, the anisotropy directions are distributed with equal probability within a cone symmetric about the *z*-axis with semiangle defined by the variable phideg. To do this, a list with an even number of elements is constructed; the first element in each pair is the name of an atlas region (i.e., grain), and the second is a three-element sublist providing the (*x, y, z*) coordinates (directional cosines) of the axis. A list of all the grain regions is obtained via a call to the GetAtlasRegions command,

set atlas_regions [GetAtlasRegions :world]

where : world refers to the Oxs_ImageAtlas: world atlas discussed above. Each call to the Texture proc (see Appendix A for the definition of proc Texture) produces a random 3-vector in the texture cone. The region names and axis directions are collated via the Tcl code


set axes { }
if {![info exists Rogue}] {set Rogue { }}
for each grain $atlas_regions {
 lappend axes $grain
 if {[lsearch −exact $Rogue $grain]>=0} {
  # Rogue grain
  lappend axes {1 0 0}
 } else {
  # Random grain
  lappend axes [Texture]
 }
}


If one wants the axis to be randomly selected from the whole unit sphere, as opposed to from inside the texture cone, then one can copy the RandomUnitVec proc from Appendix B into the MIF file, and replace the call to Texture with a call to RandomUnitVec.

In addition to setting the random vectors, this code also allows the specification of a list of “rogue” grains, where the axes are not chosen uniformly, but are peremptorily set to, say, (1, 0, 0) (i.e., the *x*-axis). The rogue grain list is set earlier in this MIF file as

set Rogue [list \#be0000 \#be85ab]

The Rogue list is a list of region names, but since the region names are determined by their color in the grain map, this is equivalent to selecting the grains by color. Many image viewers display the rgb-component values of a pixel selected by the user, so the grain map can be used to interactively select rogue grains. (As an alternative, rogue grains can be selected by position. This approach is explained below in the discussion covering Appendix B.)

Once the axes list is constructed, the anisotropy is specified by


Specify Oxs_AtlasVectorField:axes [subst {
 atlas :world
 norm 1.0
 values { $axes }
}]
Specify Oxs_UniaxialAnisotropy [subst {
 K1 $Ku
 axis :axes
}]


The second block here specifies a uniaxial anisotropy with uniform *K*1 (as defined by the variable Ku, in J/m^3^), but with spatially varying easy axis given by the vector field : axes ( i.e., Oxs_AtlasVectorField :axes).

Next in the MIF file, the exchange coupling is set by


######################
###### EXCHANGE ######
######################
set A_list { }
foreach grain $atlas_regions {
 # Intra-grain coupling
 lappend A_list \
  $grain $grain $A_intragrain
}
# default_A sets inter-grain coupling,
# A_list sets intra-grain coupling
Specify Oxs_Exchange6Ngbr [subst {
 default_A $A_intergrain
 atlas :world
 A { $A_list }
}]


In this code, the list A_list is constructed three elements at a time. The variable A_intragrain is the exchange coupling inside each grain, in J/m. In this example, this coupling is the same for all grains, but this could be varied if desired. The intragrain coupling list is used to set parameter A in the Oxs_Exchange6Ngbr specify block. The otherwise unspecified intergrain coupling is handled by the default_A parameter.

Intergranular coupling can be turned on or off by adjusting the value of A_intergrain. This may be a useful option for continuous granular media applications where decoupled grains are desirable. For fully exchanged grains as in patterned media applications, Oxs_UniformExchange may be used in place of Oxs_Exchange6Ngbr.

The remainder of the MIF file sets up an applied field, and specifies the evolver and driver to use. These are standard MIF blocks that do not involve any polycrystalline-specific features. See the OOMMF User’s Guide for details.

Appendix B provides an example using a material, such as Ni, that has cubic anisotropy. Setting up cubic anisotropy is similar to that for uniaxial anisotropy, except that instead of specifying a single easy axis, two orthogonal axes are required. (The third anisotropy axis is computed as the cross product of the other two.) Whereas in the uniaxial case a single list named axes was constructed interleaving region names and axes, in this case two lists are constructed, A_axes and B_axes. A_axes is constructed by calling the Texture proc for each grain; this produces an axis inside the texture cone defined by phideg, exactly as in the uniaxial case. The second axis, which is stored in B_axes, is constructed by picking a random vector on the unit sphere, and then crossing that vector with the associated A_axes axis element:


set bx [expr {$ay*$tmpz-$az*$tmpy}]
set by [expr {$az*$tmpx-$ax*$tmpz}]
set bz [expr {$ax*$tmpy-$ay*$tmpx}]
lappend B_axes [list $bx $by $bz]


This second axis is fully random subject to the constraint that it be orthogonal to the first axis.

Once A_list and B_list are complete, a vector field object is built with each list, and the cubic anisotropy is specified via


Specify Oxs_CubicAnisotropy [subst {
 K1 $Ku
 axisl :A_axes
 axis2 :B_axes
}]


As in the uniaxial case, rogue grain selection is built into the axis lists using a user-specified list, Rogue, of grain region names. In this example, however, the rogue list is populated by the code segment


# Map “rogue” grains to regions
for each {x y z} $RoguePos {
 lappend Rogue [GetAtlasRegionByPosition \
    :world $x $y $z]
}


Here the GetAtlasRegionByPosition command is used to correlate a spatial location (in problem coordinates, in meters) with the region (i.e., grain) containing that location. The problem coordinates may be determined using the technique described in the following section to view the anisotropy axes in mmDisp, and then using: 〈Shift〉+〈left mouse click〉 to reveal to coordinates under the mouse cursor.

### C. Visualize and Saving Anisotropy Axes

The grain anisotropy axes information may be displayed or saved through the OOMMF mmDisp viewer utility. First load a pre-simulation where the initial magnetization setting in the Driver Specify block uses the same vector field that is used to set the anistropy axis; in the uniaxial example of Appendix A that means to replace

m0 { 0 0 −1 }

with

m0 :axes

where : axes refers to Oxs_AtlasVectorField : axes.

(For the cubic anisotropy example, use : A_axes or : B_axes in place of : axes.) Once the problem is loaded into the OOMMF Oxsii application, send the driver “Magnetization” output to mmDisp, from which the axis directions can be directly viewed. One can also write these data to disk from mmDisp, but note that it will be necessary to divide each entry by the saturation magnetization *M*_s_ in order to recover the unit axes vectors.

## 3. Results With Polycrystalline OOMMF

The polycrystalline Co/Pd multilayer system is regarded as a promising candidate for patterned media applications [4–6]. Nanodots ≤ 200 nm are perpendicularly magnetized and typically switch in the single domain regime [5]. The easy axes for the material lies along the <111> and is thought to be uniaxial. Additionally, the <111> texture along the surface normal is thought to be a cause of the perpendicular magnetization [7–9]. Experimental evidence has suggested that switching in this system was triggered by local pockets of volume with dimensions on the same order as the grain size [6].

We used polycrystalline OOMMF to model a 100 nm disc, 6 nm thick, to mimic the experiment from Lau et all [1] in order to further understand the role of grain orientation. The cell dimensions are 1 nm laterally, and 3 nm along the thickness direction, giving a total of *N_x_* = *N_y_* = 100 and *N_z_* = 2 cells along the *x*, *y*, and *z* directions.

A grain map using 260 grains gives roughly the grain size of 7 nm. The uniaxial anisotropy axes of all 260 grains are uniformly distributed with a texture of 20°, that is the semiangle with respect to the surface normal (*z*-direction). The grains traverse the entire thickness of 6 nm. The numerical values used in this problem are listed in Table 1.

**Fig. 1:**
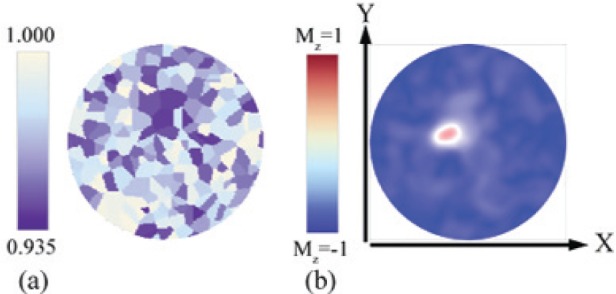
(a) Uniaxial anisotropy axis distribution across a 100 nm diameter Co/Pd-like nanodot. Color scale gives the *z* component of the uniaxial anisotropy vector. Grains in white have near perpendicular axis. Grains in purple can be up to 20° off the *z*-axis. (b) A snap shot near the coercive field of a magnetization reversal simulation using the system described in (a). The dot was initially perpendicularly magnetized along the −*z*-direction (blue). The dot is expected to be red (magnetized along the +*z*-direction) once the reversal is complete. Magnetization is primarily in-plane (white shadows) at the locations where perpendicular magnetization is weakest (corresponds to purple grains from (a). Here a localized concentration of off-axis grains is seen to trigger the onset of reversal (red spot).

Fig. 1a shows the normal component of the uniaxial anisotropy axes in the grain mosaic. Grains in white have uniaxial anisotropy axes closely aligned with the *z*-direction. Grains in purple have axes with the greatest deviation from the normal direction, with a maximum deviation of 20°. Fig. 1b shows the magnetization along the *z*-direction at the point of switching. The 100 nm dot was initially magnetized along the −*z*-direction (blue). Applying a field along the +*z*-direction causes the magnetization in the dot to rotate towards the +*z* direction (red). The white contrast within the dot means that the local magnetization is within the *xy* plane.

There is a one-to-one correspondence between the off-axis grains (purple regions, Fig. 1a) and the region of least perpendicular magnetization (regions in white Fig. 1b). Furthermore, magnetization reversal from the −*z* to +*z* direction occurs at the site of the heaviest off-axis grain concentration.

## 4. Conclusion

We have fully integrated 2D polycrystalline capability for simulating sub-100 nm magnets in OOMMF. Magneto-crystalline anisotropy magnitude and direction, as well as exchange coupling strength may be defined separately for each grain. In an example, we showed that polycrystallinity plays a significant role in the magnetization reversal mechanism in perpendicularly magnetized nanodots. Our results corroborate previous experimental observations.

## Figures and Tables

**Table 1:** Geometrical and material parameters used for simulating Co/Pd-like nanodots

*M_s_*	2000 kA/m
*K_u_*	3500 kJ/m^3^
*A*	10 pJ/m
length = width	100 nm
thickness	6 nm
*N_x_* =*N_y_*	100
*N_z_*	2

## 5. Appendix A. Sample MIF File, Uniaxial Anisotropy

**Table:**

#MIF 2.2	
#Save-path is set to current directory	#
#“stop” is the mxHxm stopping criterion	#
########################################	
set stop 0.1	
set tag UNIAXIAL	
RandomSeed 1	
# Cone angle in proc Texture (q.v.);	#
# specifies angle range for random	#
# anisotropies.	#
########################################	
Parameter phideg 10 ;# in degrees,	
## from 0 to 90, 0 is all z.	

### Input Files ###
#####################
set grain_map rect.ppm
### Output Files ###
####################
set outname [format “${tag}_phi=%g” $phideg]

###################################		
#	ROGUE GRAIN(s):	#
#Rogue is a list of colors (in hex-form		#
##rrggbb) to be set as rogue grains (which		#
#get anisotropy axis set parallel to x-axis.		#
#Leave empty or undefined to have no rogue		#
#grains.		#
###################################		
set Rogue [list \#be0000 \#be85ab]		

##########################
##### CONSTANTS #####
##########################
set pi [expr 4*atan(1.0)]
set mu0 [expr 4*$pi*1e–7]
###########################
###### MATERIALS ######
###########################

### for Co ##########################
# Saturation magnetization, A/m
set Ms 1.40e6
# Magneto-crystalline anisotropy, J/m^3^
set Ku 530e3
# Exchange coupling, J/m:
set A_intragrain 8.1e–12# ;# Intragrain
set A_intergrain 2.Oe–12# ;# Intergrain
#############################
###### SUPPORT PROCS ######
#############################
# This proc makes ellipses and circles #
###################################
proc Ellipse { Ms x y z } {
# Imports x, y, and z are each relative
# values, in range [0, 1]
set xO [expr {2*$x–1}]
set yO [expr {2*$y–1}]
if {$xO*$xO+$yO*$yO <= 1} {
return $Ms
}
return 0.0
}

#This proc generates TEXTURED unit	#
#vector, random in cone about z-axis	#
#with aperture angle 2*$phideg.	#
########################################	
set cosphirange [expr {cos($phideg*$pi/180.)}]	
proc Texture { } {	
global pi cosphirange	
set theta [expr {(2.*rand()–1.)*$pi}]	
set costheta [expr {cos($theta)}]	
set sintheta [expr {sin($theta)}]	
if {rand()<0.5}	
{ set sgn −1.0	
} else {	
set sgn 1.0	
}	
set cosphi \	
[expr {$sgn*(1−(1−$cosphirange)*rand())}]	
set sinphisq [expr {1.0−$cosphi*$cosphi}]	
if {$sinphisq>0.0} {	
set sinphi [expr {sqrt($sinphisq)}]	
} else {	
set sinphi 0.0	
}	
set x [expr {$sinphi*$costheta}]	
set y [expr {$sinphi*$sintheta}]	
set z [expr {$cosphi}]	
return [list $x $y $z]	
}	

###########################
###### ATLAS & MESH ######
###########################
# Part dimensions, in meters
set length 512e–9 ;# x extent
set width 128e–9 ;# y extent
set thick 6e–9 ;# z extent
# Cell dimensions, in meters
set xycellsize 1.Oe–9
set zcellsize 3.Oe–9

### Atlas ##############################	
#The “auto” color function sets up one	#
#region for each distinct color in the	#
#import image; the name of a region has the	#
#form #rrggbb, where rr is two hex rdigits	#
#specifying the red component (from 00 to	#
#ff), gg specifies the green component, and	#
#bb the blue component.	#
#######################################	#
Specify Oxs_ImageAtlas:world [subst {	
xrange {0 $length}	
yrange {0 $width}	
zrange {0 $thick}	
viewplane xy	
image $grain_map	
colorfunction auto	
matcherror 0.0	
}]	
# Get a comprehensive list of all regions	
# (i.e., grains)	
set atlas_regions [GetAtlasRegions :world]	
### Mesh ###	
############	
Specify Oxs_RectangularMesh:mesh [subst {	
cellsize {$xycellsize $xycellsize $zcellsize}	
atlas :world	
}]	
#################################	
###### UNIAXIAL ANISOTROPY ######	
#################################	
# This sets a random unit vector for each	#
# grain region.	#
########################################	
if {![info exists Rogue}] { set Rogue { } }	
foreach grain $atlas_regions {	
lappend axes $grain	
if {[lsearch −exact $Rogue $grain]>=0} {	
# Rogue grain	
lappend axes {1 0 0}	
} else {	
# Random grain	
lappend axes [Texture]	
}	
}	
Specify Oxs_AtlasVectorField:axes [subst {	
atlas :world	
norm 1.0	
values { $axes }	
}]	
Specify Oxs_UniaxialAnisotropy [subst {	
K1 $Ku	
axis :axes	
}]	
######################	
###### EXCHANGE ######	
######################	
set A_list { }	
foreach grain $atlas_regions {	
# Intra-grain coupling	
lappend A_list $grain $grain $A_intragrain	
}	
# default_A sets intergrain coupling,	
# A_list sets intragrain coupling	
Specify Oxs_Exchange6Ngbr [subst {	
default_A $A_intergrain	
atlas :world	
A {$A_list}	
}]	
####################################	
###### ZEEMAN (applied field) ######	
####################################	
set field 10000 ;# Maximum field (in Oe)	
Specify Oxs_UZeeman [subst {	
multiplier [expr (1./($mu0*1e4))*$field]	
Hrange {	
{000 001 10}	
}	
}]	
# H value times “multiplier” is field in A/m	
#############################	
###### DRIVER & EVOLVER ######	
#############################	
SetOptions [subst {	
basename $outname	
}]	
### Evolver ###	
###############	
Specify Oxs_CGEvolve:evolve { }	
### Driver ###	
##############	
Specify Oxs_MinDriver [subst {	
evolver evolve	
stopping_mxHxm $stop	
mesh :mesh	
Ms { Oxs_ScriptScalarField {	
atlas :world	
script_args {relpt}	
script {Ellipse $Ms}	
} }	
mO{ 0 0 −1 }	
comment {mO :axes}	
} ]	

## 6. Appendix B: Sample MIF File, Cubic Anisotropy

**Table:**

# MIF 2.2	
# Save-path is set to current directory	#
# “stop” is the mxHxm stopping criterion	#
###########################################	
set stop 0.1	
set tag CUBIC	
RandomSeed 1	
# Cone angle in proc Texture (q.v.);	#
# specifies angle range for random	#
###########################################	
# anisotropies.	
Parameter phideg 10 ;# in degrees,	
## from 0 to 90, 0 is all z.	
### Input Filess ###	
###################	
set grain_map rect.ppm	
### Output Files ###	
####################	
set outname [format “${tag}_phi=%g” $phideg]	

###########################################		
#	ROGUE GRAIN(s):	#
#	Rogue is a list of locations depicting	#
#	grain positions. Each location consists of	#
#	three values, representing x, y, and z in	#
#	simulation coordinates (meters). Leave	#
#	this list empty or undefined to have no	#
#	Rogue grains.	#
##########################################		
set RoguePos {		
391.5e–9 26.5e–9 4.5e–9		
285.5e–9 59.5e–9 4.5e–9		
}		

#######################
###### CONSTANTS ######
#######################
set pi [expr {4*atan(1.0)}]
set mu0 [expr {4*$pi*1e–7}]
#######################
###### MATERIALS ######
#######################
### for Ni ############################
# Saturation magnetization, A/m
set Ms 490e3
# Magneto-crystalline anisotropy, J/m″3
set Ku −5.7e3
# Exchange coupling, J/m:
set A_intragrain 6.9e–12 ;# Intragrain
set A_intergrain 2.Oe–12 ;# Intragrain
###########################
###### SUPPORT PROCS ######
###########################
### This proc makes ellipses and circles ###
########################################
proc Ellipse { Ms x y z } {
# Imports x, y, and z are each relative
# values, in range [0, 1]
set x0 [expr {2*$x–1}]
set y0 [expr {2*$y–1}]
if {$x0*$x0+$y0*$y0 <= 1} {
return $Ms
}
return 0.0
}

# This proc generates TEXTURED unit	#
# vector, random in cone about z-axis	#
# with aperture angle 2*$phideg.	#
########################################	
set cosphirange [expr {cos($phideg*$pi/180.)}]	
proc Texture { } {	
global pi cosphirange	
set theta [expr {(2.*rand()−1.)*$pi}]	
set costheta [expr {cos($theta)}]	
set sintheta [expr {sin($theta)}]	
if {rand()<0.5} {	
set sgn −1.0	
} else {	
set sgn 1.0	
}	
set cosphi \	
[expr {$sgn*(1−(1−$cosphirange)*rand O)}]	
set sinphisq [expr {1.0−$cosphi*$cosphi}]	
if {$sinphisq>0.0} {	
set sinphi [expr {sqrt($sinphisq)}]	
} else {	
set sinphi 0.0	
}	
set x [expr {$sinphi*$costheta}]	
set y [expr {$sinphi*$sintheta}]	
set z [expr {$cosphi}]	
return [list $x $y $z]	
}	

# This proc generates a random unit vector, #
#uniformly selected on the unit sphere. #
##################################
proc RandomUnitVec { } {
global pi
set theta [expr {(2.*rand()−1.)*$pi}]
set costheta [expr {cos($theta)}]
set sintheta [expr {sin($theta)}]
set cosphi [expr {1.0 – 2.*rand()}]
set sinphi [expr {1.0-$cosphi*$cosphi}]
if {$sinphi>0.0} {
set sinphi [expr {sgrt($sinphi)}]
}
set x [expr {$sinphi*$costheta}]
set y [expr {$sinphi*$sintheta}]
set z [expr {$cosphi}]
return [list $x $y $z]
}
##########################
###### ATLAS & MESH ######
##########################
# Part dimensions, in meters
set length 512e–9 ; # x extent
set width 128e–9 ; # y extent
set thick 6e–9 ; # z extent
# Cell dimensions, in meters
set xycellsize 1.Oe–9
set zcellsize 3.Oe–9
### Atlas ##############################
Specify Oxs_ImageAtlas:world [subst {
xrange {0 $length}
yrange {0 $width}
zrange {0 $thick}
viewplane xy
image $grain_map
colorfunction auto
matcherror 0.0
}]
# Get a comprehensive list of all regions
# (i.e., grains)
set atlas_regions [GetAtlasRegions :world]
# Map “rogue” grains to regions
foreach {x y z} $RoguePos {
lappend Rogue \
[GetAtlasRegionByPosition :world $x $y $z]
}
### Mesh ###
############
Specify Oxs_RectangularMesh:mesh [subst {
cellsize {$xycellsize $xycellsize $zcellsize}
atlas :world
}]
##############################
###### CUBIC ANISOTROPY ######
##############################

# Construct a pair of orthogonal anisotropy	#
# axes for each grain region. The third	#
# cubic anisotropy axis is automatically	#
# generated by OOMMF as c = a x b.	#
##############################################	
set A_axes {}	
set B_axes {}	
if {![info exists Rogue}] { set Rogue {} }	
foreach grain $atlas_regions {	
lappend A_axes $grain	
lappend B_axes $grain	
if {[lsearch −exact $Rogue $grain]>=0} {	
# Rogue grain	
lappend A_axes {1 0 0}	
} else {	
# Non-rogue; this grain has random	
# anisotropy axes	
# For first (“a”) axis, select unit	
# vector within phideg cone about z-axis	
set a_axis [Texture]	
lappend A_axes $a_axis	
# Construct second (“b”) axis,	
# perpendicular to “a” axis, by grabbing	
# a random vector on the sphere and	
# computing an orthogonal vector via the	
# cross product, b = a x tmp	
set ax [lindex $a_axis 0] ;# x,y,z axis	
set ay [lindex $a_axis 1] ;# components	
set az [lindex $a_axis 2]	
set tmp [RandomUnitVec]	
set tmpx [lindex $tmp 0]	
set tmpy [lindex $tmp 1]	
set tmpz [lindex $tmp 2]	
while {[expr {abs($ax*$tmpx + $ay*$tmpy \	
+ $az*$tmpz)>0.9}}] {	
# If tmp is too close to a, then a x	
# tmp may be numerically unstable.	
set tmp [RandomUnitVec]	
set tmpx [lindex $tmp 0]	
set tmpy [lindex $tmp 1]	
set tmpz [lindex $tmp 2]	
}	
# Set b = a x tmp. This is not a unit	
# vector, but the preceding check on	
# a*tmp guarantees that |b| != 0, which	
# is good enough. (b will be normalized	
# inside Oxs_AtlasVectorField:B_axes	
# below.	
set bx [expr {$ay*$tmpz-$az*$tmpy}]	
set by [expr {$az*$tmpx-$ax*$tmpz}]	
set bz [expr {$ax*$tmpy-$ay*$tmpx}]	
lappend B_axes [list $bx $by $bz]	
}	
}	

Specify Oxs_AtlasVectorField:A_axes [subst {
atlas :world
norm 1.0
values { $A_axes }
}]
Specify Oxs_AtlasVectorField:B_axes [subst {
atlas :world
norm 1.0
values { $B_axes }
}]
Specify Oxs_CubicAnisotropy [subst {
K1 $Ku
axisl :A_axes
axis2 :B_axes
}]
######################
###### EXCHANGE ######
######################
set A_list { }
foreach grain $atlas_regions {
# Intra-grain coupling
lappend A_list $grain $grain $A_intragrain
}
# default_A sets intergrain coupling,
# A_list sets intragrain coupling
Specify Oxs_Exchange6Ngbr [subst {
default_A $A_intergrain
atlas :world
A { $A_list }
}]
####################################
###### ZEEMAN (applied field) ######
####################################
set field 10000 ;# Maximum field (in Oe)
Specify Oxs_UZeeman [subst {
multiplier [expr (1./($mu0*1e4))*$field]
Hrange {
{ 0 0 0 0 0 1 10}
}
}]
# H value times “multiplier” is field in A/m
#############################
###### DRIVER & EVOLVER ######
#############################
SetOptions [subst {
basename $outname
}]
### Evolver ###
###############
Specify Oxs_CGEvolve:evolve { }
### Driver ###
##############
Specify Oxs_MinDriver [subst {
evolver evolve
stopping_mxHxm $stop
mesh :mesh
Ms { Oxs_ScriptScalarField {
atlas :world
script_args {relpt}
script {Ellipse $Ms}
} }
mO {1 0 0}
comment {mO :A_axes}
comment {mO :B_axes}
} ]
